# Toxicological Effects of Cadmium on Mammalian Testis

**DOI:** 10.3389/fgene.2020.00527

**Published:** 2020-05-26

**Authors:** Qiqi Zhu, Xiaoheng Li, Ren-Shan Ge

**Affiliations:** ^1^Department of Obstetrics and Gynecology, The Second Affiliated Hospital and Yuying Children's Hospital of Wenzhou Medical University, Wenzhou, China; ^2^Department of Anesthesiology, The Second Affiliated Hospital and Yuying Children's Hospital of Wenzhou Medical University, Wenzhou, China

**Keywords:** cadmium, reactive oxygen species, male infertility, spermatogenesis, leydig cell, testosterone

## Abstract

Cadmium is a heavy metal, and people are exposed to it through contaminated foods and smoking. In humans and other mammals, cadmium causes damage to male testis. In this review, we summarize the effects of cadmium on the development and function of the testis. Cadmium causes severe structural damage to the seminiferous tubules, Sertoli cells, and blood-testis barrier, thus leading to the loss of sperm. Cadmium hinders Leydig cell development, inhibits Leydig cell function, and induces Leydig cell tumors. Cadmium also disrupts the vascular system of the testis. Cadmium is a reactive oxygen species inducer and possibly induces DNA damage, thus epigenetically regulating somatic cell and germ cell function, leading to male subfertility/infertility.

## Introduction

Infertility rate continues to trend higher in this century and about 15% of couples are infertile. Male causes of infertility account for 40–50% (Kilchevsky and Honig, 2012). Globally, the sperm count and semen quality of men persistently trend lower (Carlsen et al., 1992; Geoffroy-Siraudin et al., 2012). The causes of male infertility are complex and the etiology of about 50% of male infertility remains unknown. Although genetic factors can explain a few percentages of male infertility, increasing environmental pollution might also contribute to the persistent increase of male infertility (Nordkap et al., 2012; Gao et al., 2015). Males are exposed to environmental pollutants throughout their life cycle, including embryonic period. Although environmental pollutants are directly exposed to the testes of adult mammals and inhibit spermatogenesis, male subfertility/infertility might also originate from fetal exposure to toxicants (Skakkebaek et al., 2001). The fetal exposure to environmental chemicals leads to reproductive tract anomalies such as cryptorchidism and hypospadias, testicular cancer, and subfertility/infertility in males, referred to as Testicular Dysgenesis Syndrome (TDS) (Skakkebaek et al., 2001). A rise of cryptorchidism and testicular cancer might significantly contribute to male subfertility/infertility (Jorgensen et al., 2011; Loebenstein et al., 2019). Although the exact mechanism for TDS is still unclear, the epigenetic regulations are involved since the fetal exposure to toxicants can persistently cause male subfertility/infertility at adulthood (Skinner et al., 2015).

Environmental pollutants include a family of heavy metals. Exposure to heavy metals has been linked to male infertility (Wirth and Mijal, 2010). One of the heavy metals is cadmium ion (Cd^2+^ or Cd). Numerous studies in animal models (mainly rodents) and growing evidence from human epidemiological research point to the adverse effects of Cd on male fertility. The present review describes Cd action and its mechanisms, including endocrine-disruption, reactive oxygen species (ROS) generation and epigenetic regulation for the etiology of male subfertility/infertility. Since male subfertility/infertility also comes from environmentally exposed fetuses, we briefly introduce the development of testis in rodents and/or humans.

## Cadmium Exposure and its Fate in Mammals

Cd is a common environmental pollutant in many industrial processes and smoking (Faroon et al., 2012). Cd is a byproduct of the production of other metals such as zinc, lead or copper, and is mainly used in batteries, pigments, coatings and electroplating, plastic stabilizers, and other applications (Faroon et al., 2012). Cd enters the food chain after contamination (Chirinos-Peinado and Castro-Bedrinana, 2020). Humans are exposed to Cd through pollutants in air, drinking water, and food (Faroon et al., 2012). Smoking is another source of Cd (Siu et al., 2009a). After smoking, the Cd content of smokers is 4–5 times higher than that of non-smokers (Takiguchi and Yoshihara, 2006). On average, the daily Cd intake of humans is 1.06 μg/kg body weight (Wan et al., 2013). Despite the lower intake of Cd, the elimination half-life of Cd is longer (~20–40 years in humans) and can accumulate in the body (Wan et al., 2013). Besides, the testis is the tissue in which Cd can accumulate in large amounts (Thompson and Bannigan, 2008). After 14 days of treatment in rats, the Cd in the testes was 100 times higher than that in the blood (Aoyagi et al., 2002). Numerous studies have shown that mammalian testes are sensitive organs against Cd (Wong et al., 2004; Takiguchi and Yoshihara, 2006; Sadik, 2008). Cd can cause male reproductive toxicity, including testicular injury (Wan et al., 2013).

## Effects of Cadmium on Spermatogenesis

### Effects of Cd on Sertoli Cells (SCs)

Mammalian testis contains two compartments, the seminiferous epithelium (SCs bind together to support spermatogenesis), and the interstitial compartment, in which Leydig cells (LCs) secrete androgen and peptide hormone such as insulin-like 3 (INSL3) to regulate the development of the male reproductive tract, the descent of testis and the spermatogenesis.

SCs play a critical role in the assembly of the testis cords during the fetal and neonatal periods (Rebourcet et al., 2014, 2016; Smith et al., 2015). When the SCs in the testis of newborn mice are eliminated, the tubule structure is lost, and subsequent development of adult Leydig cells (ALCs) in the adult testis is severely blocked (Rebourcet et al., 2014). In adult testes, SCs are essential for maintaining spermatogenesis, and elimination of the SCs in adult testes can lead to loss of germ cells (Rebourcet et al., 2014). Besides, in the fetus, SCs secrete anti-Müllerian hormone (AMH), which causes the regression of Müllerian duct (Unal et al., 2018). In the fetal life of rodents and humans, the number of SCs increases exponentially, and then slows down after birth and reaches adult levels in early puberty (Sharpe et al., 2003; O'shaughnessy et al., 2007; Guo et al., 2020; Tan et al., 2020).

Cd affects SC development during fetal and neonatal periods (Table 1). A single intraperitoneal injection (ip.) of low doses of Cd to rats on GD12 down-regulates the expression of SC genes (*Dhh* and *Fshr*), although this does not affect its number (Li et al., 2018). Exposure to Cd (1–2 mg/kg, sc) in pregnant and lactating rats can cause vacuolation of SCs and loss of germ cells in adult seminiferous epithelium (Bekheet, 2011). Cd inhibits proliferation and induces apoptosis and DNA damage of immature SCs in the piglet testis (Zhang et al., 2018). Cd inhibits the interaction between neonatal SC and gonocyte via p38 MAPK signaling in the SC-gonocyte co-culture system *in vitro* (Yu et al., 2008).

**Table 1:** Action of cadmium on testicular cells.

**Species**	**Cell**	**Action**	**References**
**SERTOLI CELL**			
Pig	Sertoli cell	DNA damage (+), apoptosis (+)	Zhang et al., 2018
Rat	Sertoli cell	*Dhh* and *Fshr* expression (–) Ultrastructure alteration (+), Cytoplasmic vacuolation (+), Cytoskeleton disarrangement (+) BTB disruption (+)	Hew et al., 1993; Haffor and Abou-Tarboush, 2004; Wong et al., 2005; de Souza Predes et al., 2011; Xiao et al., 2014; Li et al., 2018; Zhu et al., 2018
Human	Sertoli cell	BTB disruption (+)	Xiao et al., 2014
Mouse	Sertoli cell	Mitochondrial alteration (+)	Bizarro et al., 2003
**LEYDIG CELL**			
Rat	Fetal Leydig cell	Steroidogenic gene expression (–) Testosterone synthesis (–) *Insl3* expression (–)	Hu et al., 2014; Li et al., 2018
Rat	Adult Leydig cell	Leydig cell development (–) Leydig cell number (–) Testosterone synthesis (–) Leydig cell volume (–) Cytoplasm vacuolization (+) Leydig cell tumor (+) Leydig cell regeneration (–)	Mckenna et al., 1996; Waalkes et al., 1997, 1999; Biswas et al., 2001; Gunnarsson et al., 2004; Blanco et al., 2007, 2010; Cupertino et al., 2017; Wu et al., 2017; Mahmoudi et al., 2018; Tian et al., 2018
Mouse	Adult Leydig cell	Steroidogenic gene expression (–) Testosterone secretion (–) Leydig cell number (–) Leydig cell cytoplasm alteration (+) Leydig cell tumors (+)	Hu et al., 2014; Mahmoudi et al., 2018
**GERM CELLS**			
Rat	Spermatogenesis	Spermatogonia number (–), Massive germ cell death (+)	Cupertino et al., 2017; Mahmoudi et al., 2018
Human	Sperm	Motility (–)	Vine, 1996
Rat	Sperm	Sperm motility (–), Sperm count (–), *In vitro* fertilization rate (–), Early embryonic development (–)	Zhao et al., 2017; Mahmoudi et al., 2018

*BTB, blood-testis barrier; (–) or (+), inhibition or stimulation*.

During puberty and adulthood, SCs form a blood-testis barrier (BTB) to support spermatogenesis. Spermatogenesis takes place in the seminiferous tubules, which are formed by SCs. Spermatogenesis invovles the self-renewal and proliferation of spernatogenia, and then differentiation, cell cycle progression from type B spermatogonia to preleptotene spermatocytes outside of the BTB, cell cycle progression from zygotene and pachytene to diplotene spermatocytes, a transition of round spermatids to elongated spermatids and then spermatozoa via spermiogenesis, and spermiation (Wu et al., 2019). In the adult testis, SCs play key roles in supporting the self-renewal and differentiation of spermatogonia into mature sperm (Su et al., 2018). SCs provide a basic link between the interstitium and the seminiferous tubule (Su et al., 2018). Therefore, SCs play essential roles in spermatogenesis.

Adult SCs are the target of Cd (Table 1). Exposure of rats to Cd of 1 mg/kg daily by gavage for 28 days can cause severe ultrastructure changes in adult SCs (Haffor and Abou-Tarboush, 2004). Rats exposed to a single dose of Cd (3 μmol/kg) show vacuolation in the SC cytoplasm and irregular chromatin condensation in late spermatids (de Souza Predes et al., 2011). Exposure to Cd by inhalation for 28 days can cause severe mitochondrial changes in SCs of adult mice (Bizarro et al., 2003). Molecular biology findings indicate that Cd perturbs the cytoskeleton of SC actin by disrupting F-actin organization in human SCs at 0.5–20 μM after altering the expression of actin regulatory proteins *Arp3* and *Eps8 in vitro* (Xiao et al., 2014).

### Effect of Cd on BTB Formed by SCs

The BTB in a mammalian testis consists of a specialized junction between adjacent SCs near the basement membrane in the seminiferous tubule (Wu et al., 2019). The BTB is the target of Cd (Table 1). Cd induces the disruption of BTB in rodent models. Cd attacks BTB by inducing defragmentation of actin filaments of SCs in rodents (Wong et al., 2005) and humans (Xiao et al., 2014). The mechanistic finding demonstrates that Cd disturbs BTB in the rat testis *in vivo* by up-regulating transforming growth factor β3 (TGF-β3), which in turn activates p38 MAPK signaling (Lui et al., 2003; Wong et al., 2004). Interestingly, Cd also activates the JNK pathway at the same time to up-regulate α2-macroglobulin to counteract its adverse effects because JNK specific inhibitor can aggravate Cd-induced damage on BTB (Wong et al., 2005), indicating the JNK signaling is the protective mechanism in SCs after Cd treatment. Cd treatment to SCs at 5–10 μM for 8 h can disrupt SC tight junction assembly by down-regulating the expression of occludin and urokinase plasminogen activator without causing any apparent cytotoxicity and T can protect it (Chung and Cheng, 2001).

Focal adhesion kinase (FAK) is a non-receptor protein tyrosine kinase to regulate BTB (Wan et al., 2014). FAK regulates tight junction proteins (e.g., occludin and ZO-1) in the rat testis (Siu et al., 2009b,c). Cd can down-regulate FAK expression (Siu et al., 2009c). The knockdown of FAK in SCs with a functional tight junction can protect SCs from Cd-induced disruption (Siu et al., 2009b). This indicates that Cd targets FAK to regulate BTB.

### Effect of Cd on Sperm Development

Cd affects sperm development (Table 1). Rats exposed to a single dose of (0.67–1.1 mg/kg) of Cd for 7 days show disorganization of the seminiferous epithelium (Cupertino et al., 2017). After 28 days of oral administration of Cd (5 mg/kg) for 28 days, rat's sperm count, motility, and viability decline (Nna et al., 2017). When rats are exposed to Cd (0.2 mg/kg, sc) for 15 days, the seminiferous tubules of their testes are disarranged, the number of germ cells decreases (Jahan et al., 2014). Adult male rats have significantly damaged seminiferous tubules after 56 days of exposure to Cd (1.15 mg/kg, i.p) (Leite et al., 2013). Cd (3 mg/kg, sc, once a week) exposure to rats for 4 weeks also contract seminiferous tubules and deplete germ cells and increase multinucleated giant cells (Rajendar et al., 2012).

### Effects of Cd on Mature Sperm Function

Cd affects mature sperm function (Table 1). After *in vitro* treatment with human and mouse sperm, Cd remarkably reduces sperm motility and progressive motility (Zhao et al., 2017). Short-term treatment of Cd (30 min) will not influence sperm motility, but significantly reduces the *in vitro* fertilization rate to egg and delays early embryonic development in mice, suggesting that Cd works epigenetically (Zhao et al., 2017). Cd also lowers human sperm motility and forward motility (Zhao et al., 2017).

## Human Epidemiological Studies of Cadmium

The effects of Cd on human fertility have been reviewed in several papers (de Angelis et al., 2017; Kumar and Sharma, 2019). Evidence from epidemiological studies supports the positive correlation between Cd and male subfertility/infertility. Fifty cases of hypospadias and healthy control boys are analyzed for the association with serum heavy metal concentrations oncentrations (Sharma et al., 2014). Serum concentrations of Cd in hypospadias boys are significantly higher (Sharma et al., 2014). Comparing the serum and semen Cd levels of 60 infertile adult males in Nigeria (40 oligospermia and 20 azoospermia) with 40 normal spermia controls, the data have shown that Cd and FSH levels of these infertile patients are significant higher (Akinloye et al., 2006). Infertile couples (501 cases) in Rockville of the United States show higher Cd levels in their blood, indicating that Cd has reproductive toxicity at environmentally relevant levels (Buck Louis et al., 2012). Men with varicocele usually show increased accumulation of Cd in the testicular blood system, and the percentage of sperm cell apoptosis in their testes also generally increases (Benoff et al., 2004). Meta-analysis with high-quality studies can provide superior evidence. Zhang et al. collect 11 research articles (including 1093 infertile subjects and 614 controls) and perform a meta-analysis and find that a high level of Cd in semen causes male infertility (Zhang et al., 2019). De Franciscis et al. investigated fifty healthy men and found that blood Cd concentrations were positively associated with a reduction of sperm motility and teratozoospermia (de Franciscis et al., 2015). He et al. measured the urinary levels of oxidative stress markers, semen quality, and urinary levels of three heavy metals including arsenic, Cd and lead in 1020 men and indicate that that higher levels of urinary arsenic, Cd and lead are negatively associated with semen quality and positively associated with increased oxidative stress markers (He et al., 2020).

Cd also causes endocrine-disrupting effects on males. Xu et al. reported in a study of 2,286 men (aged 18 years and older) that there is a negative association between blood Cd levels with total T and sex hormone-binding globulin (SHBG) (Chen et al., 2016). Kresovich et al. examined the associations in males in National Health and Nutritional Examination Survey (NHANES) data from 1999 to 2004 for blood Cd and SHBG and found that blood Cd was positively associated with SHBG (Kresovich et al., 2015). These studies indicate that Cd might be negatively associated with total or free T levels.

## Mechanisms of Cadmium-Mediated Action

### The Endocrine-Disrupting Effects

LCs are endocrine cells in the testis. There are two populations of LCs in rodents, fetal LC (FLC) and adult LC (ALC). On GD12 in mice or GD14 in rats, a set of fetal Leydig cell (FLC) progenitors are differentiated into FLCs after the regulation by SC-secreted factors, such as desert hedgehog (Yao et al., 2002) and platelet-derived growth factor (Brennan et al., 2003) and aristaless-related homeobox (Miyabayashi et al., 2013). In rodents, FLCs double cell number and persist in the testis until birth (Barsoum and Yao, 2009). FLCs undergo apoptosis and gradually disappear although some persist in the adult testis (Kerr and Knell, 1988; Shima et al., 2015). FLCs play an essential role in the development of the male reproductive tract by synthesizing androgen (mainly, T) and insulin-like 3 (INSL3). Androgen promotes the development of both the internal and external genitalia of the male fetus and descent of testis (Ye et al., 2017). In male mammals, androgen promotes the Wolffian duct to develop into the epididymis, vas deferens, and seminal vesicles (Ye et al., 2017). INSL3 promotes the initial descent of the testis by regulating gubernaculum shortening (Adham et al., 2000). The shortening of the gubernaculum draws the testes from the kidney position across the abdomen to the entrance of the inguinal canal (Emmen et al., 2000).

ALCs emerge as progenitor Leydig cells in the late second week after birth in mice and rats, and express some LC steroidogenic enzymes, such as cytochrome P450 side-chain cleavage enzyme (*Cyp11a1*), 3β-hydroxysteroid dehydrogenase (*Hsd3b, Hsd3b6* in the mouse and *Hsd3b1* for the rat ALC) isoforms, and cytochrome P450 17α-hydroxylase/17,20-lyase (*Cyp17a1*) but lack the last-step T synthetic enzyme, 17β-hydroxysteroid dehydrogenase 3 (*Hsd17b3*) (Ge and Hardy, 1998; Wang et al., 2003). Progenitor Leydig cells transit into the immature stage, in which immature Leydig cells (ILCs) express *Hsd17b3*, but they have a high expression of 5α-reductase 1 (*Srd5a1*) during 4–5th weeks (Ge and Hardy, 1998; Wang et al., 2003). Then, they finally mature into ALCs, which synthesize T with full capacity, after the silence of *Srd5a1* (Ge and Hardy, 1998; Wang et al., 2003). Besides, ALCs also express luteinizing hormone (LH) receptor (*Lhcgr*) to receive signaling from pituitary-secreted LH, and high-density lipoprotein receptor (*Scarb1*) and steroidogenic acute regulatory protein (*Star*) for cholesterol transport.

As mentioned above, FLCs secrete two important hormones, T and INSL3, to stimulate the development of the male reproductive tract and the descent of the testis. Perinatal exposure to environmental toxicants can cause TDS (Wang et al., 2019). Cd affects the development and function of FLCs (Table 1). Pregnant rats receiving a single dose of Cd (0.25, 0.5, and 1.0 mg/kg, i.p.) can remarkably decrease T synthesis of the fetal testis, lower FLC number, down-regulate the expression of FLC genes (*Lhcgr, Scarb1, Star, Cyp11a1, Hsd3b1*, and *Cyp17a1*) of male offspring (Li et al., 2018) and it also shortens the anogenital distance, an androgen-dependent process, of male offspring (Li et al., 2018). In the testis of rats that are perinatally (during gestation and lactation) exposed to Cd and the development of ALCs is delayed, with an increase in the number of ILCs (*Srd5a1*-expressing cells), and decrease in cAMP/PKA signaling and down-regulation of T steroidogenic enzymes (Tian et al., 2018).

Cd also affects the development and function of ALCs (Table 1). Adult male mice exposed to Cd (0.015 g/L in drinking water) at 1, 3, 6, and 12 months have significantly decreased ALC cell volume (Blanco et al., 2007). Male mice exposed to Cd-containing food (about 1–2 g per animal) for half a year have lower T secretion and lower expression of *Star, Cyp11a1*, and *Cyp17a1* (Hu et al., 2014). Mature male mice fed with 0.015 g/L Cd in drinking water for 0.25, 0.5, 1, and 1.5 years exhibit cytoplasmic vacuolization in ALCs, reduction of ALC number and formation of LC tumors (Blanco et al., 2010). After 7 days of exposure to a single dose (0.67–1.1 mg/kg) of Cd, serum T levels in rats are significantly reduced (Cupertino et al., 2017). Adult male rats exposed to Cd (sc) have increased PGF2α and decreased serum T levels and down-regulated *Star* (Gunnarsson et al., 2004). Adult male rats exposed to Cd (0.5 or 1.0 mg/kg, i.p. single dose) also have significantly delayed LC regeneration, lower T levels and down-regulated expression of *Lhcgr, Scarb1, Star, Cyp11a1, Hsd3b1, Cyp17a1*, and *Hsd17b3* (Wu et al., 2017). Adult rats receiving a single dose of Cd (0.45 mg/kg, sc) have a significantly low HSD3B1 and HSD17B3 activity and serum T levels and accessory sex organ weight (Biswas et al., 2001). Further *in vitro* studies have shown that Cd also lowers T synthesis and DNA integrity of LCs (Liu et al., 2013).

Cd concentration-dependently lowers cAMP and down-regulates the expression of dihydrolipoamide dehydrogenase in R2C tumor LCs (Zhang et al., 2011). Primary LCs that are exposed to 10, 20, and 40 μM of Cd for 24 h also show the increase in DNA damage and lower T secretion (Yang et al., 2003). Besides, Cd also induces LC tumors (Waalkes et al., 1999) and disrupts vascular cells (Leite et al., 2015).

### Cadmium Induces ROS Production

There is increasing evidence that the mechanism by which Cd mediates impaired male fertility is related to the production of ROS in the testes. ROS is composed of hydroxyl, peroxyl, and hydroperoxyl radicals, superoxide, nitric oxide, and nitrogen dioxide. The homeostasis of ROS is maintained by the production of ROS and the antioxidant system. This disruption of homeostasis leads to oxidative stress, which hinders the development and function of sperm and somatic cells or induces apoptosis (Morielli and O'flaherty, 2015). Cd induces ROS generation in the testis. Cd (6.5 mg/kg) exposure to adult rats for 5 days increases oxidative stress, including increased peroxidation and nitric oxide and decreased GSH level, catalase, superoxide dismutase (SOD), glutathione peroxidase, and glutathione reductase, thus up-regulating the expression of pro-apoptotic protein BCL-2-associated-X-protein (*Bax*) and tumor necrosis factor-α and down-regulating the expression of the anti-apoptotic gene (*Bcl2*) in the testis, leading to a decrease of cell proliferation (Elmallah et al., 2017). Rats that are exposed to Cd (1.5 mg/kg) for 13, 25, and 39 days have the increase in ROS production and possess the reduction of the diameter of the seminiferous tube, the decrease of the number of spermatogonia, SCs, and LCs, and the decrease in sperm motility and count, as well as the inhibition of T synthesis (Mahmoudi et al., 2018). Cd exposure to adult mice (1 mg/kg, i.p.) for 5 and 8 weeks increases lipid peroxidation and decreases SOD, catalase, and peroxidase in the testis, leading to an increase in sperm abnormality and decrease in sperm count (Acharya et al., 2008).

Exposure of Cd (40 mg/L) to rats for 30 days significantly lowers testis and seminal vesicle weights and decreases serum T levels and sperm count as well as sperm motility by increasing ROS levels and suppressing catalase and SOD activity (Amara et al., 2008). Cd exposure to adult male rats after a single dose (2 mg/kg, sc) for 24 h induces ROS generation and decreases SOD and catalase activity in the testis, thus disrupting the BTB and vitamin C can antagonize Cd-induced BTB damage by inhibiting TGF-β3 activation and p38 MAPK phosphorylation (Chen et al., 2018). Cd exposure to rats increases ROS level and decreases glutathione peroxidase and superoxide dismutase activity, thus leading to the down-regulation of *Star* and *Hsd3b1* and *Hsd17b3* and lowering secretion of T (Sen Gupta et al., 2004). Cd exposure to rats after i.p. 0.025 mg/kg/day for 15 days induces ROS production and lowers SOD, catalase, glucose-6-phosphate dehydrogenase, and glutathione-S-transferase activity in the mitochondrion (Pandya et al., 2012). This exposure causes a remarkable reduction of the expression of LC steroidogenic enzymes (*Hsd3b1* and *Hsd17b3*) and T synthesis (Pandya et al., 2012). Rats exposed to 0.2 mg/kg Cd (sc) for 5 days have significantly high lipid peroxidation and low catalase, peroxidase, SOD, and glutathione reductase activity in the testis (Jahan et al., 2014). Rats exposed to Cd (3 mg/kg, sc, once a week) for 4 weeks display shrunken tubules and depletion of germ cells, increase of multinucleated giant cells, and degeneration of LCs after inducing significantly high ROS levels and low SOD and catalase activity and low amount of GSH (Sugiura et al., 2005; Rajendar et al., 2012). Cd (30 μmol/kg) exposure to rats remarkably increases lipid peroxidation and formation of H_2_O_2_ in LCs and decreases glutathione reductase and catalase activities after 12 h treatment and induces LC tumors later (Koizumi and Li, 1992). Cd, after being administered in a single dose (1 mg Cd/kg, i.p.) into male mice, causes interstitial hemorrhages, LC death, and numerous atypical mitoses of the spermatocytes after 3 and 6 months (Selypes et al., 1992).

The *in vitro* system also shows that Cd induces ROS production in various testicular cells. *In vitro* SC-germ cell co-culture shows that Cd induces ROS and decreases GSH, thus causing cytochrome c release, caspase-3 activation and SC apoptosis (Khanna et al., 2011). Cd exposure to rat R2C tumor LC cells at 10–160 μM for 24 h also causes mitochondrial damage and lowers *Star* expression level and then inhibits steroid secretion, possibly by increasing ROS levels and decreasing SOD2 activity (Yan et al., 2019). Cd down-regulates the expression of *Star, Cyp11a1*, and *Hsd3b1* by inhibiting cAMP/PKA/ERK1/2 and PKC signaling after inhibiting of dihydrolipoamide dehydrogenase activity in R2C tumor LCs (Ji et al., 2015). Primary rat ILCs exposed to Cd have a reduced mitochondrial membrane potential and increased ROS MAPK-extracellular-regulated kinase activity, increased cell death, and a decreased transcription of *Hsd3b1* (Khanna et al., 2016). Cd concentration-dependently inhibits hCG- and db-cAMP-stimulated T production *in vitro* (Laskey and Phelps, 1991). After 48 h of Cd treatment with 0.03 mmol/kg Cd, it can induce DNA damage and apoptosis in rat testes (Xu et al., 1996).

Cd exposure to mouse TM3 tumor LCs also reduces LC viability and increases cell apoptosis after increasing ROS production and JNK phosphorylation and c-Jun expression, then activates apoptosis-related proteins, cleaved-caspase 3 and cleaved-PARP, and decreases BCL2. These effects can be reversed by antioxidant N-acetyl-L-cysteine and JNK inhibitor (Lu et al., 2019). Exposure of Cd to TM3 tumor LCs decreases SOD2 and GSH contents by targeting the Nrf2/ARE signaling pathway, thereby decreasing T production (Yang et al., 2019).

All antioxidants, including vitamin C (Acharya et al., 2008; Pandya et al., 2012; Chen et al., 2018), vitamin E (Acharya et al., 2008; Jahan et al., 2014), Fragaria ananassa extract (Elmallah et al., 2017), Ficus religiosa (Jahan et al., 2014), cyanidin-3-O-glucoside (Yan et al., 2019), N-acetyl-L-cysteine (Khanna et al., 2016; Lu et al., 2019), sulphoraphane (Jahan et al., 2014), green tea (Mahmoudi et al., 2018), quercetin (Nna et al., 2017), Paullinia cupana (Leite et al., 2013), alpha-tocopherol (Rajendar et al., 2012), selenium (Bekheet, 2011), and zinc (Villanueva et al., 2005; Burukoglu and Baycu, 2008) can partially and completely antagonize the Cd-mediated effect, suggesting that the major pathway of Cd is ROS induction.

### Epigenetic Regulation of Cd

Although interesting epigenetic effects are observed in the germline after Cd treatment (Zhao et al., 2017), environmentally induced epigenetic changes related to infertility are described in somatic cells (such as SCs and LCs), which support spermatogenesis. Rats exposed to Cd (1, 2, or 4 mg/kg/day) on days 3–7 after birth causes abnormal DNA methylation on day 70 after birth and increases sperm apoptosis, and exhibits a degradation of seminiferous tubules (Zhu et al., 2011). Exposure of rats to Cd-contaminated soil for 1 year leads to Cd accumulation and increases the genome-wide methylation status and the expression of DNA methyltransferase (Dnmt 3a/3b) in the testis, suggesting epigenetic changes (Nakayama et al., 2019). Treatment of mouse TM3 LC cell line with Cd can down-regulate the expression of DNA methyltransferase (Singh et al., 2009), also indicating an epigenetic regulation and a possible cancer formation.

In other cell types, Cd can also up-regulate the expression of two oncogenic epigenetic regulators, viz. protein arginine methyltransferase 5 and the polycomb repressive complex 2 member enhancer of Zeste homolog 2, which in turns lead to an increased global level of symmetric dimethylarginine, H4R3me2s and H3K27me3, and it can also induce global DNA hypomethylation due to a decrease in DNA methyltransferase expression. This may be involved in the epigenetic regulation of Cd-mediated cancer formation (Ghosh et al., 2019).

## Conclusion

**Figure 1:**
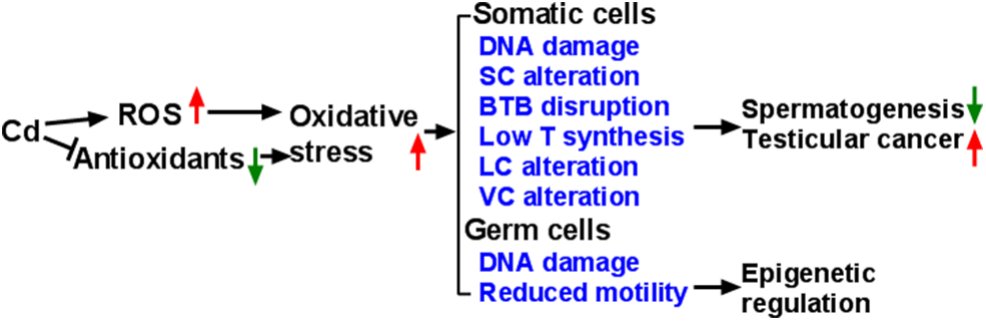
Illustration of Mechanism of Cadmium (Cd) induces reactive oxygen species (ROS) generation and down-regulates endogenous antioxidants, thus leading to oxidative stress, which in turn causes abnormal development and function of testicular cells. Eventually, Cd exposure leads to abnormal spermatogenesis, testicular cancer, and damaged sperms. SC, Sertoli cell; BTB, blood-testis barrier; T, testosterone; LC, Leydig cell; VC, vascular cell.

It is now generally accepted that the mammalian testes are very sensitive to Cd, leading to changes in the testicular biochemical function. Cd induces the production of ROS and reduces the activity of antioxidative enzymes, thereby causing oxidative damage to the testes (Figure 1). Cd also epigenetically regulates testicular cells. Cd induces vacuolation and destruction of seminiferous epithelium, abnormal changes in SC ultrastructure. After damaging SC function, Cd disrupts BTB, increases the seminiferous tubule permeability and disrupts spermatogenesis. Cd disrupts the development and function of LCs by inducing DNA damage and apoptosis, and down-regulating the expression of the steroidogenesis-related genes, resulting in decreased T secretion. Much work is required to explore the events that occur during Cd-induced testicular injury.

## Author Contributions

QZ and XL wrote this review. R-SG edited the manuscript.

## Conflict of Interest

The authors declare that the research was conducted in the absence of any commercial or financial relationships that could be construed as a potential conflict of interest.
